# DNA damage induces GDNF secretion in the tumor microenvironment with paracrine effects promoting prostate cancer treatment resistance

**DOI:** 10.18632/oncotarget.3040

**Published:** 2014-12-10

**Authors:** Roland M. Huber, Jared M. Lucas, Luis A. Gomez-Sarosi, Ilsa Coleman, Song Zhao, Roger Coleman, Peter S. Nelson

**Affiliations:** ^1^ Division of Human Biology, Fred Hutchinson Cancer Research Center, Seattle, WA, USA; ^2^ Division of Clinical Research, Fred Hutchinson Cancer Research Center, Seattle, WA, USA

**Keywords:** microenvironment, paracrine, resistance, prostate cancer, treatment

## Abstract

Though metastatic cancers often initially respond to genotoxic therapeutics, acquired resistance is common. In addition to cytotoxic effects on tumor cells, DNA damaging agents such as ionizing radiation and chemotherapy induce injury in benign cells of the tumor microenvironment resulting in the production of paracrine-acting factors capable of promoting tumor resistance phenotypes. In studies designed to characterize the responses of prostate and bone stromal cells to genotoxic stress, we found that transcripts encoding glial cell line-derived neurotrophic factor (GDNF) increased several fold following exposures to cytotoxic agents including radiation, the topoisomerase inhibitor mitoxantrone and the microtubule poison docetaxel. Fibroblast GDNF exerted paracrine effects toward prostate cancer cells resulting in enhanced tumor cell proliferation and invasion, and these effects were concordant with the expression of known GDNF receptors GFRA1 and RET. Exposure to GDNF also induced tumor cell resistance to mitoxantrone and docetaxel chemotherapy. Together, these findings support an important role for tumor microenvironment damage responses in modulating treatment resistance and identify the GDNF signaling pathway as a potential target for improving responses to conventional genotoxic therapeutics.

## INTRODUCTION

Prostate cancer-related mortality is primarily caused by metastatic disease, a disease stage that initially shows high response rates to androgen deprivation therapy (ADT) but subsequent progression to a clinical state termed castration resistant prostate cancer (CRPC) [1-3]. Current treatment options for CRPC include DNA damaging regimens (genotoxic chemotherapy and radiation) but their impact on overall survival remains limited [4] and CRPC is almost invariably fatal. DNA damaging regimens are not selective to prostate cancer cells but also affect other normal cellular components that exist in the tumor microenvironments (TME) of organs and tissues. The spectrum of benign cells comprising the TME varies between tumors located at the primary site and those that have disseminated to distant metastatic sites, but generally includes fibroblasts, vascular cells, nerve cells, and various components of the immune system. DNA damage to TME cells can lead to the activation of secretory programs which in turn influence the growth and resistance behavior of tumor cells [5].

In studies designed to characterize transcriptional alterations in primary prostate fibroblasts following DNA damage, we previously identified more than 40 transcripts that encode secreted proteins with the potential for exerting paracrine effects on adjacent tumor cells [6]. This cohort of secreted proteins included Glial Cell line-Derived Neurotrophic Factor (GDNF). GDNF was first described as a trophic factor for dopaminergic neurons of the rat midbrain [7]. Additional trophic effects on various other neuronal subpopulations have subsequently been described: GDNF acts as a morphogen in kidney development and it is a key component regulating the spermatogonial stem cell niche and the cellular proliferation of spermatogonial stem cells [8, 9]. These effects are signaled via a receptor multimer that comprises the RET transmembrane-receptor in conjunction with GNDF-family co-receptors, mainly the *alpha 1* type (GFRA1). Activated GDNF/GFRA1/RET complexes signal via the SRC/ERK, RAS/MAPK and the PI3K/PKB pathways in a cell context-dependent manner [8]. A RET independent pathway that is responsive to GDNF via GFRA1 and SRC family kinases (SFK) has been reported thereby uncoupling an obligate requirement for RET expression in mediating GDNF effects in target cells [10].

GDNF has been shown to be elevated in prostate cancer reactive tumor stroma where it is hypothesized to contribute to tumor growth and invasion [11]. Sympathetic and parasympathetic innervation of the prostate is linked to poor clinical outcome in prostate cancer patients by inducing early phase tumor development and late stage tumor dissemination [12]. GDNF has well established functions as a neuro-trophic factor and as a neurite guidance cue [13], but whether GDNF affects the innervation pattern of prostate cancers after genotoxic treatment is not known, and such an effect would likely be evident long after exposures, a scenario not directly relevant to clinical realities in metastatic disease. However, GDNF could have comparable trophic effects on prostate cancer cells and stromal cells as seen in neurons and other tissues, with acute effects on prostate cancer growth and treatment resistance.

The fact that GDNF is up-regulated in prostate tumor stroma in various settings and given the well-established function of GDNF as a trophic factor in multiple different tissues types, we hypothesized that GDNF secretion by the TME upon DNA damage could actively influence prostate cancer cellular responses and/or growth properties, and thereby modify their sensitivity to cytotoxic, cytostatic or pathway-directed therapeutics. We report here that genotoxic treatments induce GDNF secretion in both prostate fibroblasts and bone fibroblasts, which consequently induces prostate cancer cell proliferation and treatment resistance. These effects correlate with the expression levels of GFRA1 receptor in the cancer cells. GDNF stimulation activates the SRC/ERK pathway and induces RB and E2F1 mediated transcriptional changes. GDNF has treatment counter-acting effects in prostate cancer cells which may limit the efficacy of DNA damaging strategies for treating localized and advanced prostate cancers.

## RESULTS

### DNA damage induces GDNF secretion in stromal cells comprising the prostate tumor microenvironment

**Figure 1:**
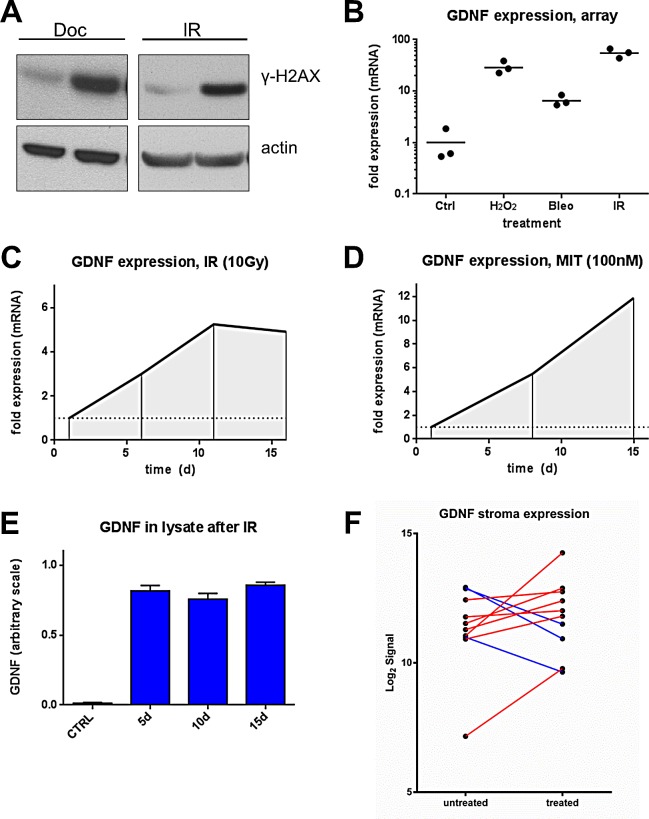
DNA damage induces GDNF expression in human prostate fibroblasts (A) Western blot probing for DNA damage marker y-H2AX post Docetaxel (DOC) (50 nM) and irradiation (IR) (10 Gy) in PSC27 cell lysates. (B) Gene expression microarray data of GDNF in human prostate stromal cells treated with hydrogen peroxide (H_2_O_2_), Bleomycin (Bleo) and irradiation (IR) on log scale. qPCR data showing up-regulation of GDNF after (C) irradiation between 6 and 16 days post treatment and after (D) mitoxantrone (MIT) treatment between days 7 and 15 post treatment. (E) ELISA assay measuring GDNF protein in cell lysates (Ly) 5d, 10d and 15d after DNA damage induced by irradiation (10 Gy) compared to non-irradiated control (CTRL). (F) GDNF transcript level changes measured by microarrays in micro-dissected CaP stroma after treatment with DOC and MIT in 10 paired patient samples.

We previously profiled the gene expression alterations in prostate fibroblasts following DNA damage and identified a spectrum of highly induced transcripts of which a subset encoded secreted proteins including GDNF [6]. To confirm and quantify these findings in independent experiments, we exposed PSC27 prostate myofibroblasts [14] to agents known to cause DNA damage: 0.6 mM H_2_0_2_, 10 μg/ml bleomycin, 100 nM mitoxantrone and 10 Gy radiation (IR). We confirmed the induction of DNA damage in the treated cells by assessing the phosphorylation status of serine 139 on H2A histone family, member X (γ-H2AX), indicative of DNA double strand breaks (Fig 1A). We also treated PSC27 fibroblasts with the microtubule poison docetaxel, a chemotherapeutic widely used in the treatment of advanced prostate cancer. Exposure to 50 nM docetaxel also resulted in DNA damage with γ-H2AX phosphorylation detected at levels roughly equivalent to that observed with IR (Fig 1A), a finding concordant with previous findings showing docetaxel induces DNA damage, though indirectly via replication-mediated double strand breaks [15, 16]. Using microarray-based methods to quantitate gene expression alterations we determined that *GDNF* expression increased substantially (> 6-fold) regardless of the agent used to induce DNA damage (Fig 1B). To determine the temporal pattern of GDNF up-regulation after DNA damage, we measured *GDNF* transcript levels by qRT-PCR from one to 16 days post treatment and observed a gradual increase in *GDNF* transcripts over time, from 2.5-fold at 5 days to 5-fold by 16 days after IR, and from 3.5-fold to 11.5-fold after mitoxantrone treatment (p<0.001) (Fig 1C, D). However, when analyzing the amount of intracellular GDNF protein in prostate fibroblasts after these treatments by ELISA, protein induction to above detection limit was found as early as five days post exposure, and the concentration did not further increase over time despite further increases in *GDNF* transcript levels (Fig 1E), suggesting that GDNF protein may be secreted. We also compared *GDNF* transcript levels by microarray analysis in micro-dissected cancer-associated stromal tissue before and after exposure to chemotherapy in men with prostate cancer enrolled in a neoadjuvant clinical trial combining mitoxantrone and docetaxel [17, 18]. In the majority of cases analyzed (7/10), *GDNF* transcripts were elevated after therapy in these paired clinical samples (Fig 1 F) consistent with the findings in cultured prostate fibroblasts.

### DNA damage-induced GDNF secretion exerts autocrine effects on prostate fibroblasts

**Figure 2:**
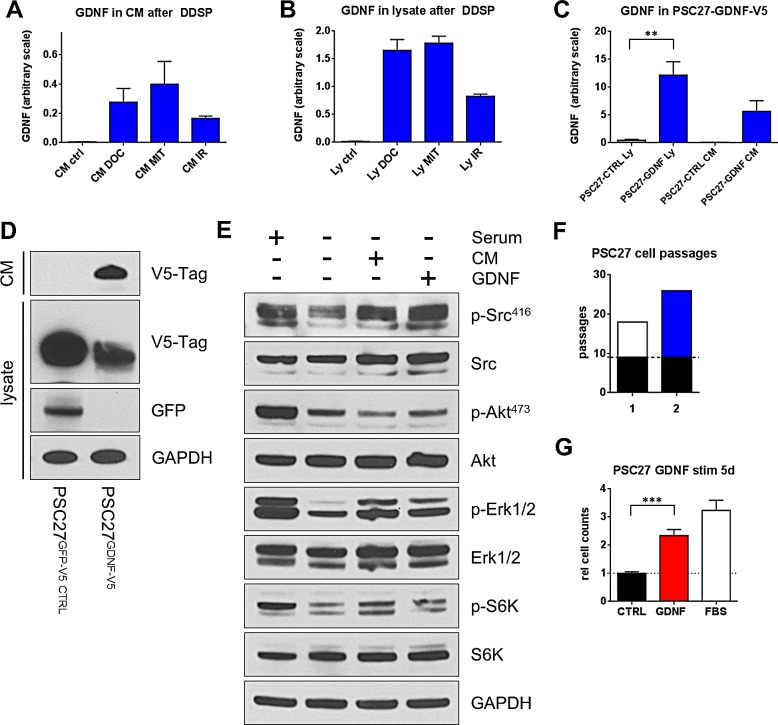
DNA damages induces GDNF secretion producing autocrine effects in prostate fibroblasts GDNF protein levels measured by ELISA in (A) conditioned medium (CM) and (B) cell lysates (Ly) of PSC27 prostate stromal fibroblasts after DNA damage by treatment with Docetaxel (DOC; 50 nM), Mitoxantrone (MIT; 100 nM), or irradiation (IR; 10 Gy) 15d after treatment. (C) GDNF specific ELISA measuring GDNF levels in Ly and CM of virally transduced PSC27 cells and (D) western blot analysis of both CM and Ly of the same cell line. (E) Western blot analysis of signaling pathways in PSC27 cells after stimulation with 100 ng/ml hrGDNF or CM of GDNF over-expressing cells. (F) Parental PSC27 (black, 9 passages) were transduced with TurboGFP (white, #1) or GDNF-V5 (blue, #2) and passaged to replicative exhaustion. Passage numbers are shown as absolute counts. (G) PSC27 cells stimulated with hrGDNF for 5 days, relative cell counts are shown.

As GDNF exerts its biological function as a secreted ligand, we next analyzed the effect of DNA damaging treatments on GDNF secretion. As the tumor microenvironment is comprised of tumor cells and benign constituents including fibroblasts, we determined whether GDNF exhibits paracrine and/or autocrine effects toward this abundant benign cell type. We treated PSC27 fibroblasts with three DNA damaging regimens and measured GDNF secretion to the culture medium (CM) by ELISA. We were unable to detect GDNF in the CM of control cells. However, after treatment with IR, docetaxel, or mitoxantrone, GDNF was measureable in the CM at physiologically relevant concentrations (> 5 ng/ml) (Fig 2A). The same effect was observed when analyzing cell lysates of PSC27 fibroblasts after treatment, with substantial GDNF protein measurable in all three treatment conditions (Fig 2B). Collectively, these results demonstrate that clinically relevant treatment regimens induce the transcription, translation and secretion of GDNF in prostate fibroblasts.

GDNF can activate multiple down-stream signaling cascades via its known receptors RET and GFRA1 [8]. To determine if GDNF acts in an autocrine/paracrine loop via one of these pathways, we analyzed their activation state in PSC27 cells before and after exposure to exogenous GDNF. To generate a model isolating the effect of GDNF amongst the amalgam of secreted factors induced by DNA damage, we generated PSC27 cells over-expressing GDNF by viral transduction. These PSC27-GDNF-V5 cells showed substantially increased levels (>10-fold) of GDNF in cell lysates and in the conditioned CM compared to PSC27-EGFP-V5 control cells (Fig. 2C D). We treated PSC27 fibroblasts with 100 ng/ml purified human recombinant GDNF (hrGDNF) as well as conditioned medium from PSC27-GDNF-V5 cells. Stimulation with GDNF led to the activation of SRC kinase which is known to be an effector kinase downstream of GFRA1. Additionally, we found the ERK pathway to be activated after GDNF stimulation but not AKT, indicating that GDNF activates selective down-stream pathways in prostate fibroblasts (Fig 2E). GDNF exposure also resulted in the activation of S6 kinase indicating that GDNF stimulation could have pro-proliferative and pro-survival effects. To test this possibility, we analyzed the replicative potential and proliferation rates of cells exposed to GDNF. PSC27 cells are primary prostate fibroblasts with limited replicative potential. These cells can be grown for 18 to 19 passages before undergoing replicative exhaustion and/or cell senescence after which they no longer proliferate. PSC27 control cells (PSC27-EGFP-V5) underwent replicative growth arrest after 18 passages, whereas PSC27 cells expressing GDNF (PSC27-GDNF-V5) underwent replicative growth arrest after 26 passages (Fig 2F). Stimulation of low passage PSC27 cells with intact proliferative potential showed that GDNF also significantly enhanced their proliferation rate (> 2-fold, p<0.001) (Fig 2G). Together, these data show that cancer therapeutics, such as IR or chemotherapy, to which tumor microenvironment cells are often exposed, induce GDNF secretion in these tumor microenvironment cells which then stimulates the stromal cells in an autocrine/paracrine loop via the SRC/ERK pathway.

### Bone marrow fibroblasts secrete GDNF following DNA damage but lack autocrine GDNF responses

**Figure 3:**
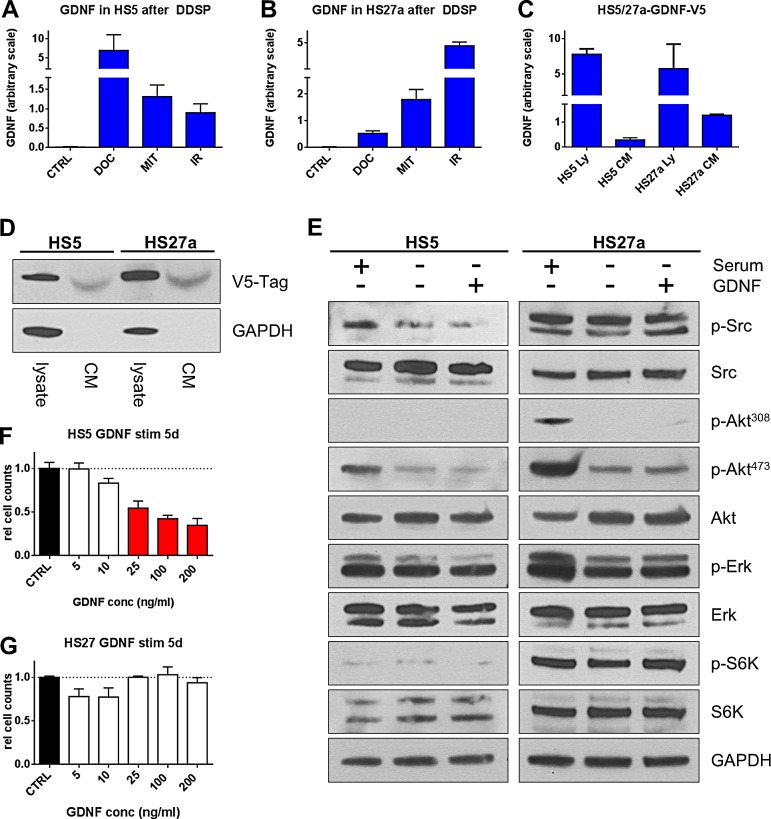
Bone fibroblasts induce GDNF following DNA damage but lack autocrine signaling GDNF protein levels measured by ELISA in cell lysates of (A) HS5 and (B) HS27a human bone stromal cells after DNA damage by treatment with Docetaxel (DOC; 1 nM), Mitoxantrone (MIT; 100 nM), or irradiation (IR; 10 Gy) 15d after treatment. (C) ELISA analysis of GDNF expression in cell lysate and secretion in to CM of bone stromal cells virally transduced to over-express GDNF tagged with a V5-epitope. (D) Western blot analysis of GDNF expression (Ly) and secretion in to the conditioned medium (CM) of HS-GDNF-V5 cells. (E) Bone stromal cells HS5 and HS27a were stimulated with full serum (FBS) or 100 ng/ml hrGDNF and signaling pathways were analyzed by western blot. Cell counts for (F) HS5 and (G) HS27a bone stormal cells stimulated with increasing concentrations of GDNF after 5d of culturing. Significant changes (p≤0.05) are shown as red bars.

DNA damaging regimens are used focally in the case of radiation to the primary site or discrete metastatic foci, regionally in the case of bone-directed radioisotopes such as RAD-223, and systemically when using genotoxic chemotherapeutics for the treatment of CRPC which often metastasizes to bone [19]. To determine if DNA damaging regimens also induce GDNF in benign cells residing in the bone marrow niche, we measured GDNF expression after treatment with IR (10 Gy), docetaxel (1 nM) and mitoxantrone (100 nM) in two bone stromal cell lines (HS5 and HS27a, [20]). In both cell lines, GDNF was below detection limit in control settings. However, exposure to IR, docetaxel, or mitoxantrone strongly induced GDNF protein production as measured by a GDNF specific ELISA (Fig 3A, B). To isolate GDNF effects from the spectrum of other damage-induced factors, we generated GDNF over-expressing cell lines for HS5 and HS27a cells (Fig 3C, D). The proliferation rates of the GDNF over-expressing bone fibroblast lines did not differ from the control cells (data not shown). Subsequent analysis of the signaling pathways which are known to be down-stream of GDNF and which were activated in prostate fibroblasts, such as SRC and ERK phosphorylation, did not show any activation in HS5 or HS27a cells (Fig 3E). There were also no changes in AKT phosphorylation status or activation of down-stream S6 kinase, indicating that the bone stromal cells, unlike prostate fibroblasts, are not sensitive to autocrine/paracrine stimulation with GDNF, despite the fact that they do substantially increase GDNF expression after genotoxic damage. While exposure to GDNF did not produce changes in SRC, ERK and S6K activation patterns, increasing GDNF concentrations (25-200 ng/ml) did have an anti-proliferative effect on HS5 fibroblasts when compared to control conditions or to cells stimulated with low concentrations of GDNF (5-10 ng/ml; p<0.05) (Fig 3F). HS27a fibroblasts did not show significant changes in cell growth or proliferation in these assays (Fig 3G).

### GDNF stimulation of prostate cancer induces cell growth and activates mitotic signaling pathways

Prostate and bone stromal cells substantially increased GDNF expression and secretion following genotoxic damage, indicating that GDNF produced in the tumor microenvironment could exert effects toward prostate cancer cells at the primary site and at the predominant site of metastasis. We therefore sought to determine if GDNF influenced prostate cancer phenotypes of growth, invasion, and resistance to cancer therapeutics.

**Figure 4:**
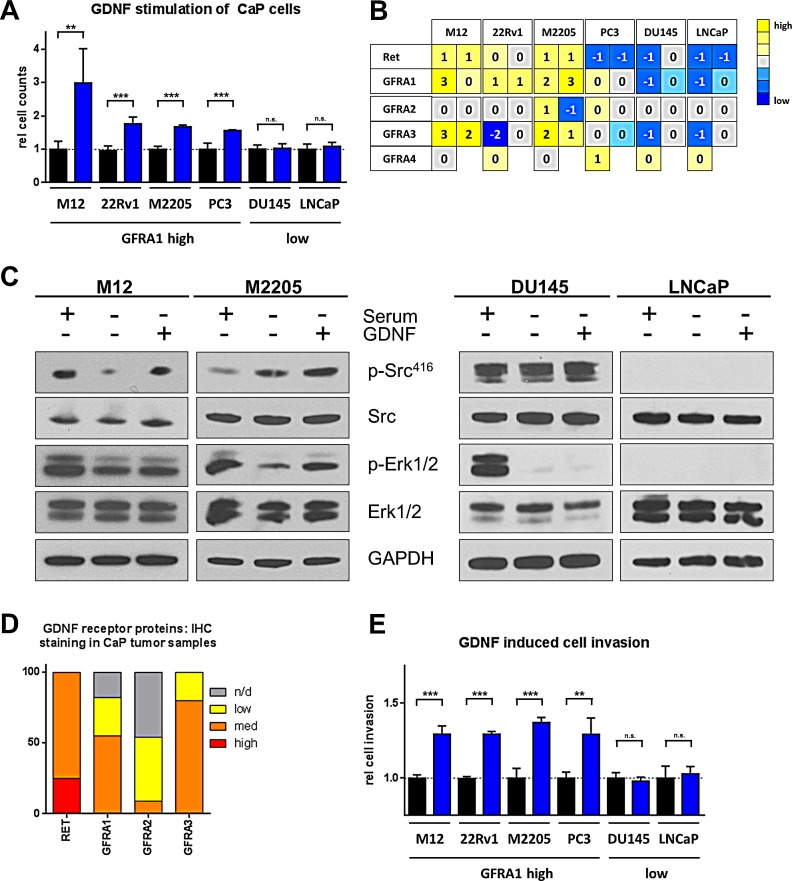
Prostate cancer cells respond to GDNF stimulation and activate SRC and ERK pathways (A) Cell proliferation assay counting viable cells after 5d of stimulation with 5-10 ng/ml hrGDNF in serum free conditions. (B) Transcript level analysis for RET receptor and GFRA-familiy members in CaP cell lines, one array probe per box are shown. (C) Western blot analysis of signaling pathway activation in GDNF responsive cells (M12 and M2205) and GDNF insensitive cells (DU145 and LNCaP). (D) Protein staining for GDNF family receptors and RET from the Protein Atlas data base in CaP patient samples. (E) Cell invasion assay using 100 ng/ml of hrGDNF as chemo-attractant in serum free conditions.

To determine the sensitivity of prostate cancer to GDNF, we exposed a spectrum of prostate cancer cell lines to GDNF and monitored cell proliferation over a period of 3-5 days. Four cell lines (M12, 22Rv1, M2205, PC3) showed significant increases in cell numbers and proliferation when exposed to GDNF (1.5- to 3-fold) while two cell lines (DU145, LNCaP) where insensitive to GDNF stimulation (Fig 4A). GDNF signaling is mediated via the cell surface receptors RET and GFRA1, with distinct downstream pathway connectivity [8]. To determine if the growth response of the epithelial cells corresponded to the expression pattern of either of these receptors, we analyzed the expression levels of RET as well as of the GFR-alpha receptor family genes. GFRA1 is the predominant GDNF receptor, but contributions from other family members have been reported [8]. There was a clear correlation between GDNF sensitivity of the prostate cancer cell lines and GFRA1 expression levels which separated the four sensitive lines from the non-responsive lines (Fig 4B). The cells with the strongest response also had the highest levels of RET, excepting PC3 cells which only showed limited expression of RET while still being sensitive to GDNF, indicating that GFRA1/SRC signaling could be the predominant pathway involved. There was no noticeable correlation between GDNF sensitivity and expression of any of the other GFR-alpha family members (Fig 4B). This expression pattern of high *GFRA1* and moderate or absent *RET* and *GFRA2-4* was also found in the GDNF sensitive PSC27 cells (Supplementary Fig S1A). Next, we analyzed the activation status of the GFRA1/SRC/ERK pathway in the sensitive and insensitive cell lines. Responsive cells showed increased SRC phosphorylation and elevated levels of activated ERK while insensitive cells did not (Fig 4C). Stimulation with GDNF did not induce AKT activation in any of the prostate cancer lines analyzed (data not shown), consistent with the findings in prostate and bone stromal cells (Fig 2E; Fig 3E). Together, these findings indicate that prostate cancer cells expressing *GFRA1* respond to GDNF stimulation with the activation of the SRC/ERK pathway and increased mitotic rates. The cell lines with the strongest response to GDNF also had the highest expression levels of RET, indicating that this effect could be enhanced by co-expression of both receptors. Adding GDNF neutralizing antibodies (NAb) to conditioned medium of stromal cells with induced DDSP reduced the pro-proliferative effect in CaP epithelial cells (Supplementary Fig S1B).

We analyzed publicly available data at *The Human Protein Atlas* data repository [21, 22] to determine the expression patterns of RET and GFRA-family members in clinical prostate cancer. The majority of prostate cancers expressed GFRA1 (82%) and all tumors showed RET expression (Fig 4D), indicating that a majority of localized prostate cancers are potentially sensitive to GDNF.

GDNF is a major regulator of directed neurite growth [23], indicating that it also could have effects on the migratory and invasive behavior of prostate cancer. To test this hypothesis, we analyzed the effect GDNF has as a chemo-attractant in trans-well invasion assays in the same cell lines used in the proliferation study. The four cell lines expressing *GFRA1* (M12, 22Rv1, M2205, PC3) showed significant increases in migration and invasion after 24h (130-135%, p < 0.01), whereas the two cell lines with low or absent *GFRA1* (DU145, LNCaP) did not exhibit any significant changes (Fig 4E). To control for the possibility that the difference in invasiveness could be caused by GDNF pro-proliferative effects, we measured changes in cell numbers after 24 hours of GDNF exposure and found no significant differences in this time frame (Supplementary Fig S1C). Overall we observed a growth promoting and pro-invasive influence of GDNF toward prostate cancer cells. These effects correlated with the expression of *GFRA1,* but not necessarily with *RET* or *GFRA2-4*. Cells responding to GDNF showed activation of the SRC/ERK pathway.

### GDNF enhances prostate cancer resistance to genotoxic chemotherapy

**Figure 5:**
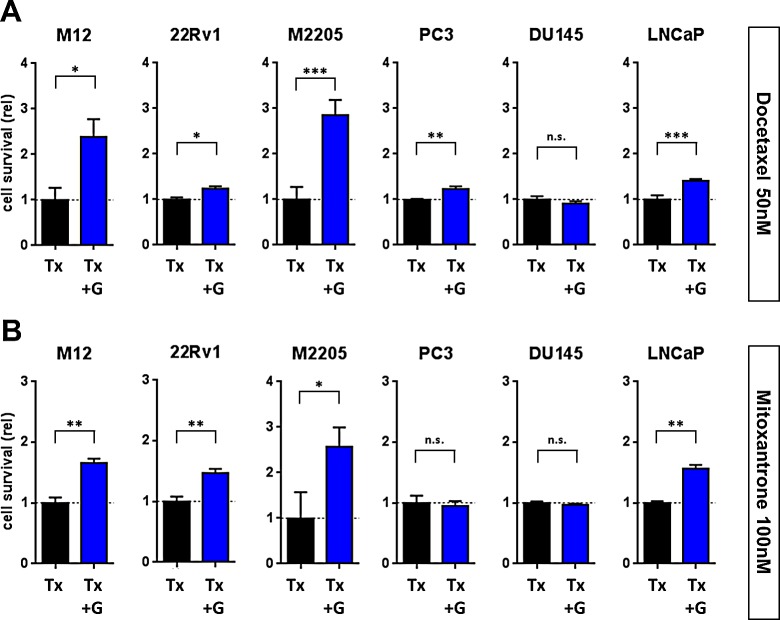
GDNF promotes tumor cell resistance to genotoxic chemotherapy Epithelial CaP cells were stimulated with 100 ng/ml hrGDNF in serum free conditions and treated for 5d with (A) 50 nM Docetaxel or (B) 100 nM Mitoxantrone. Cell numbers and viability were analyzed and are shown as normalized values compared to Tx w/o GDNF stimulation as baseline.

The changes in cell proliferation and the activation of the SRC/ERK pathway in prostate cancer cells upon stimulation with GDNF indicated that GDNF could also have an effect on the survival of prostate cancer in the context of cancer-directed therapeutics. We stimulated prostate cancer cells with 100 ng/ml hrGDNF and co-treated them with either 50 nM of docetaxel or 100 nM mitoxantrone. After an incubation period of 5 days we analyzed the number of viable cells comparing treatments with or without GDNF exposure. In the context of docetaxel treatment, the addition of GDNF enhanced the viability of all cell lines tested, excepting DU145 (Fig 5A). The DU145 effects are consistent with a lack of proliferation and invasion responses to GDNF and reflect the absence of RET and GFRA1 receptors that would mediate these responses (Fig 4B). In the context of mitoxantrone, GDNF enhanced the cell viability to the greatest extent in those cell lines with the highest *GFRA1* expression, M12 and M2205 (1.7-fold and 2.6-fold, p<0.05) whereas the survival of cells with low to absent GFRA1, such as DU145, was not enhanced (Fig 5B). However, the survival of LNCaP cells which express very low levels of *RET* and *GFRA1*, was enhanced by GDNF despite not having shown increases in proliferation or invasion. The mechanism underlying this response has not been established. These findings indicate that GDNF secretion upon DNA damage could directly influence the response rate and effectiveness of prostate cancer therapeutics and thereby contribute to acquired treatment resistance.

### GDNF induces distinct gene expression programs in prostate cancer cells and prostate fibroblasts

**Figure 6:**
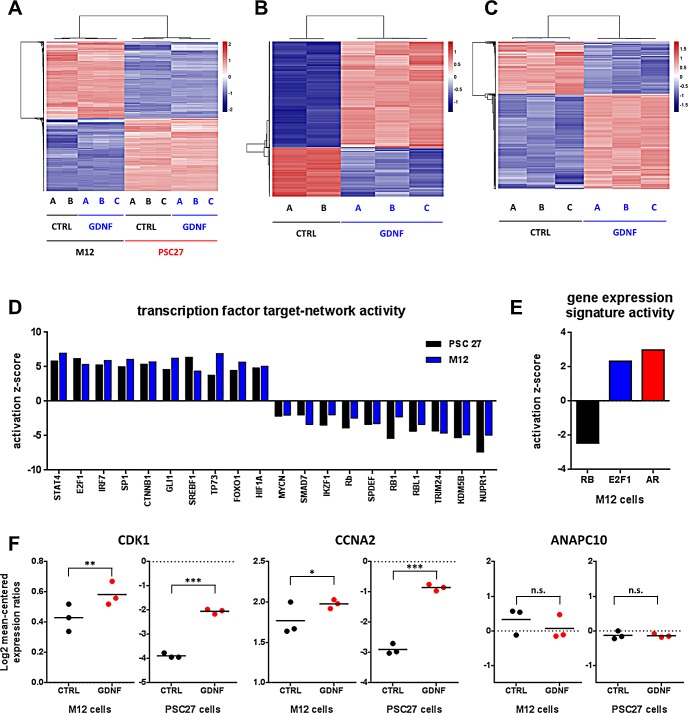
GDNF induces gene expression changes via the activation of transcription factor networks Heat map profiles of gene expression changes upon GDNF stimulation in (A) epithelial and stromal prostate cancer cells, (B) in M12 epithelial cells alone, and (C) in PSC27 PPFs alone. (D) Activation scores for transcription factor target gene groups after GDNF stimulation in PSC27 (black) and M12 (blue) cells. (E) Activation scores for RB, E2F1 and AR target gene groups after GDNF stimulation in epithelial CaP cells. (F) Gene expression changes of known E2F1 and AR target genes with enhancer modules regulated by GDNF stimulation.

GDNF produced substantial changes in the phenotypes of both prostate epithelial and stromal cells. GDNF is known to regulate cellular behavior across a spectrum of tissues by indirectly influencing gene expression [8, 24, 25]. To identify the gene expression programs influenced by GDNF we used whole-genome microarrays to quantitate transcript abundance levels before and after GDNF exposure. We isolated RNA from M12 prostate epithelial cells and PSC27 prostate fibroblasts stimulated with 100 ng/ml hrGDNF for 48h in chemically defined culture medium. In M12 cells, 763 transcripts increased and 291 decreased following exposure to GDNF (FDR q≤0.01). In PSC27 fibroblasts, 735 transcripts increased and 383 decreased (FDR q≤0.01). Comparisons across the two cell lines identified 95 up-regulated and 25 down-regulated genes common to both gene sets (FDR q≤0.01). We performed unsupervised sample clustering based on these expression profiles. The primary attribute driving the sample grouping was cell type (Fig 6A) followed by absence (control) or presence of GDNF stimulation (Fig 6B, C) in both cell lines.

We next sought to identify any regulatory networks and known interaction pathways that would provide an indication of the collective effects of GDNF in these cell types. Using the *Ingenuity Pathway Analysis* algorithms [26] we found a strong and highly significant correlation with the disease areas of ‘Cancer’ and ‘Muscular Disorders’ with strong enrichment of pro-survival and pro-proliferation functions. These effects could be narrowed down to ‘cell cycle progression’, ‘cellular growth and proliferation’, and ‘cell death and survival’ networks thereby confirming that the biochemical and phenotype changes we observed *in vitro* were also reflected in changes of global gene expression. Using these findings as an internal control for the quality and relevance of the transcript profiles, we next aimed to identify transcriptional regulators mediating the effect of GDNF stimulation on the target cells.

### GDNF regulates genes comprising RB1-, E2F1- and AR-target gene clusters

To determine how GDNF stimulation of epithelial and stromal cells leads to the observed changes in overall gene expression and identify which transcription regulators possibly mediate this effect, we matched the GDNF-associated gene expression profiles to the target-gene patterns of known transcription factors. Ingenuity algorithms produce positive or negative activation z-scores that indicate if the target genes of a specific regulatory entity, in this case a transcription factor, correlates with activation or inhibition. We grouped the transcription factors based on the combined activation score in fibroblasts and epithelial cells with cutoffs at 2 > z > −2 and a p-value of p < 0.05 corresponding to those transcription factor target groups that are strongly induced or repressed (Fig 6D). The cohort of transcription factors with reduced activity included several members of the retinoblastoma family (Fig 6D). A previous study identified the retinoblastoma (RB1) tumor suppressor as a regulator of prostate cancer progression in a sub-set of CRPC cases [27]. RB1 loss was shown to activate the transcription factor E2F1, which subsequently augmented androgen receptor (AR) activity. The combined effect of cell cycle deregulation via RB1 and E2F1 and of AR re-activation was hypothesized to promote prostate cancer progression to castration resistance. Interestingly, E2F1 targets were among the most significantly activated genes after GDNF stimulation (Fig 6D). The initial combined analysis of carcinoma cells and fibroblasts did not identify AR targets as significantly altered. However, when analyzing the data for the cancer cells independently, the reduction of the RB gene expression signature (z = −2.46) and activation of the E2F1 signature (z = 2.34) were confirmed, and activation of the AR signature (z = 2.99) was significant (Fig 6E). Prior studies have reported that RB1 levels affect the AR occupancy of select gene enhancer sequences [27]. We evaluated the transcript levels for genes putatively affected by RB1-AR interactions and found that several genes, including CDK1 and CCNA2, were up-regulated following GDNF exposure while others (ANAPC10) were not (Fig 6F), indicating that the RB1-AR effect only targets a subset of the target genes, potentially in a tissue specific manner. Together, these data indicate that GDNF stimulation is associated with reduced RB activity and enhanced E2F1 and AR target gene expression in a subset of prostate carcinoma that are receptive to GDNF signaling by virtue of RET and/or GFRA1 receptor expression.

## DISCUSSION

Studies of tumor responses to genotoxic treatments often demonstrate initial cytotoxic effects and tumor regressions, but subsequent tumor progression with accelerated rates of tumor cell repopulation. Several mechanisms appear to underlie treatment resistance, making targeted intervention to circumvent resistance challenging and multi-dimensional [28]. Treatment pressures, such as chemo- or radio-therapy, will select for pre-existing neoplastic sub-clones with elevated resistance levels [29]. Subsequent cell populations derived thereof will retain these characteristics and gradually increase treatment resistance with repeated selection/therapy cycles. Additionally, exposure to treatment can induce or increase the expression levels and activity of adaptive resistance mechanisms, such as the up-regulation of MDR genes, activation of DNA repair mechanisms or switching of the predominant metabolic pathways [30]. These effects render the tumor cells less sensitive to treatment conditions and can act in concert with the effect of sub-clonal selection.

Treatment failure and tumor repopulation are not exclusively mediated by cancer cell intrinsic mechanisms. Paracrine interactions with the tumor stroma and micro-environment have been described with resistance-enhancing effects [6], adding an additional layer of complexity. The cancer-TME interaction can be mediated via the ECM and direct cell-to-cell contacts for short range interactions, or via soluble biologicals acting across multiple tissue layers. The tumor micro-architecture influences critical aspects of treatment sensitivity, such as intra-tumoral pressure/edema, innervation or vascularization. Multiple levels of treatment resistance that include cell intrinsic mechanisms, paracrine interactions between tumor and stroma, and the micro-architecture of the TME could be substantially affected by the induction of GDNF following genotoxic damage.

GDNF is up-regulated and secreted by stromal cells that reside in the prostate and the bone marrow, indicating that both primary lesions and metastases could be stimulated by GDNF in various different treatment settings that span localized irradiation of the primary lesion and systemic chemotherapy for widely metastatic disease. Specifically, GDNF stimulation increases the proliferation rate of prostate cancer cells. This effect correlates with the expression level of *GFRA1*, but not with those of *RET*, and signaling seems to occur via the GFRA1/SFK pathway. The majority of prostate cancer patient samples analyzed show GFRA1 protein expression, indicating that increased cell proliferation upon GDNF secretion in the TME and therefore expedited tumor repopulation and clonal outgrowth could contribute to treatment failure in a considerable proportion of prostate cancer cases. The fact that GDNF reduces the sensitivity of prostate cancer to commonly used treatment regimens could further enhance this effect by increasing the pool of actively proliferating cells during and after genotoxic treatment cycles. ERK signaling is known to interfere with multiple pathways regulating apoptosis [31] and blocking Caspase-3 activation [32] as well as inducing DNA damage repair genes in CaP cells [33], offering direct molecular links between GDNF and the observed phenotypes. However, increased proliferation, elevated drug tolerance, and modified apoptosis signaling are likely to be gradual variations of a single continuous effect, the activation of pro-mitotic signaling within the target cell.

Tumor repopulation by treatment-resistant circulating tumor cells (CTC) has been suggested as one mechanism for rapid recurrence and treatment failure [34]. GDNF acted as a chemo-attractant to prostate cancer cells in our experiments. Consequently, as bone stromal cells secrete GDNF upon DNA damage, they could act as an attractant to viable CTCs in the circulation, an effect that would add to the pro-survival responses of GFRA1/SFK activation by promoting the homing of resistant prostate cancer cells to a microenvironment with augmented pro-growth and resistance-inducing properties. In the prostate itself, GDNF-induced chemotaxis of prostate tumor cells could induce cell migration to areas of sustained viability and niches with more favorable growth conditions after genotoxic insults. This effect could be even further enhanced by GDNF inducing re-innervation and re-vascularization of the particular niche after radiation therapy.

GDNF is best described as a neuro-trophic factor with strong chemotactic potential on neurite growth and directionality [13] and it maintains the spermatogonial stem cell niche in rodents in a regulated, pro-proliferative state [9]. A local source of GDNF in the tumor stroma could therefore act as an additive factor in TME remodeling and survival after therapy. Prostate fibroblasts showed strong responses to GDNF by increasing their proliferative rate and delaying cell senescence *in vitro*. A combined effect of increased stromal cell survival and enhanced TME remodeling post-treatment could have considerable influences on the dynamics of tumor treatment resistance and repopulation by maintaining a favorable niche for tumor cells to survive and proliferate within, particularly in the case of radiation therapy where sufficient time for vast tissue remodeling and re-innervation is available. As autonomic innervation has been shown to correlate with disease progression in prostate cancer [12], GDNF could exert adverse indirect effects via it's known neurotrophic activities.

Overcoming acquired treatment resistance has the potential to arrest disease progression and considerably improve overall outcomes in patients with metastatic cancers. DNA damage induces a DNA damage secretory program (DDSP) comprised of tens to hundreds of paracrine-acting proteins that are capable of influencing tumor growth, survival, and resistance to therapy. While it is attractive to consider specific therapeutics directed toward inhibiting the effects of GDNF in the context of standard cancer therapeutics, the DDSP is comprised of additional factors that have the potential to promote resistance phenotypes. Understanding the upstream regulatory mechanisms that contribute to the secretion of GDNF and other DDSP components could provide strategies for suppressing the DDSP more broadly, and thereby limit the treatment counter-acting effects exerted by a damaged tumor microenvironment.

## MATERIALS AND METHODS

### Western Blot

Cells were washed once in 1x PBS before lysis in 1% SDS (Fisher, BP166), 1% NP-40 (Sigma, 74385), 2% Tween-20 (Fisher, BP337), 1.5M urea (Fisher, BP169) in PBS. Lysates were collected with a cell scraper and heated to 95°C for 5min. DNA was sheared using a small gauge syringe. For sub-cellular fractionation, cells were lysed in 1% NP-40, 10% Glycerol (Fisher, BP229), 2mM EDTA (Fisher, BP120), 137mM NaCl (Fisher, BP358), 20mM TrisHCl (Promega, H5123) in H_2_0, pH adjusted to 8.0, and nuclei pelleted by centrifugation. Nuclei were then lysed in the high urea lysis buffer and treated as described above. All lysis buffers were supplemented with phosphatase inhibitors (phosSTOP, Roche, 04 906 837 001) and stored at −20°C.

Electrophoresis was performed on 4-12% gradient gels (Novex, NP0321) using MES SDS buffer (Invitrogen, NP0002-02). Transfer was performed on a semi-dry transfer unit (Amersham, TE 70) using Tris/CAPS buffer (BioRad, 161-0778) and 0.2μm nitrocellulose membranes (Novex, LC2000). Membranes were pre-blocked in 5% non-fat milk, 1% BSA (Sigma, A3294-100G) in PBS-T, and labeled with primary or secondary antibody in 1% BSA PBS-T. Primary antibodies used were: V5-Tag (Invitrogen, R960-25), p-Src (Cell Signaling, 2101S), Src (Cell Signaling, 2110S), p-Akt (S473) (Cell Signaling, 4058S), p-Akt (T308) (Cell Signaling, 4056S), Akt (Cell Signaling, 9272S), p-Erk1/2 (T202/Y204) (Cell Signaling, 4370), Erk1/2 (Cell Signaling, 9102), p-S6K p70 (Cell Signaling, 9204S), S6K p70 (Cell Signaling, 9202S), GAPDH (GeneTex, GTX627408), TurboGFP (Thermo Scientific, PA5-22688), γ-H2AX (GeneTex, GTX11174), and actin (Cell Signaling, 4970S).

Secondary antibodies were Goat derived IgG coupled to HRP (Pierce, 31460, 31402, 31430). Chemiluminescent substrate (Pierce, 34080) was used for visualization on light sensitive film (Thermo Scientific, 34093).

### GDNF ELISA

For conditioned cell culture medium analysis, the medium was replaced with serum free medium for 4-6 days, collected and concentrated using centrifugal filter units (Millipore, UFC900324). For lysate analysis, the same lysates were used as for western blots (see above). ELISA was performed according to the manufacturer's instructions (Promega, G7621) in at least triplicate dilution series. Concentrations were calculated based on the GDNF reference standard curve and adjusted for background signal with 0.25 on the arbitrary scale corresponding to approximately 100 ng/ml of GDNF in the analyzed sample.

### qRT-PCR

The following sequences were used for GDNF specific primers: fwd. TCC CAT TCA GAG AAC CTT GGC AGT; rev. ACC TGC TTG TGG TGT GTA GGT GAT. Expression levels were normalized to the expression signal of GAPDH in all experiments. qRT-PCR reactions were performed using ‘Power SybrGreen’ on an Applied Biosystems (Foster City, CA) 7900HT Fast Real-Time PCR System according to manufacturer's instructions. Reaction cycling conditions consisted of a 10′ incubation at 95°C followed by 40 cycles of 95°C for 15 seconds with a one minute extension phase at 60°C. Product disassociation curves were generated using the machines default conditions.

### Cell Counting and Viability Assay

The cells were counted and analyzed for viability with a ‘ViCELL XR’ automated cell counter (Beckman Coulter) and analyzed with the ‘ViCELLXR 2.04.004’ software according to manufacturer's instructions. In short, cells were suspended by trypsination and collected in RPMI 1640 containing 10% FBS to stop the enzymatic reaction. The cells were then analyzed in the cell counter which uses trypan blue exclusion to determine sample cell viability. Cells were treated as indicated in the text. For GDNF neutralization assays the following antibodies were used: mouse anti GDNF (R&D, Cat # MAB212, 5ug/ml) and Goat anti GDNF (R&D, Cat # AF-212-NA, 2ug/ml). Conditioned medium of PSC27 cells irradiated with 10 Gy was collected. Neoplastic prostate epithelial cells were stimulated in a mix of serum free medium and conditioned medium 1:1 in the presence (NAb) or absence (CTRL) of GDNF NAb.

### Cell Invasion Assay

Cell invasion was analyzed using the ‘CultureCoat 96 well Low BME Cell Invasion Assay’ (CULTUREX, Cat# 3481-096-K) according to the manufacturer's instructions. The cells used in the assay were starved for 48h in serum free culture medium prior to seeding. Serum free medium was used as a control condition for base line invasiveness, 10% FBS RPMI 1640 was used as a positive control, and 100ng/ml human recombinant GDNF (hrGDNF, R&D Systems, 212-GD-050) in serum free RPMI was used in the experimental set. The assay plates were read on a ‘Synergy 2’ plate reader (BioTek) and analyzed with ‘Gen5’ software (BioTek).

### Cell Line Generation and Culture Conditions

Prostate cancer cell lines M12, 22Rv1, M2205, PC-3, DU145 and LNCaP were obtained from the American Type Culture Collection. PSC27 prostate myofibroblast cells were generated by Dr. Beatrice Knudsen [14]. Hs5 cells and Hs27a cells were kindly provided by Dr. Beverly J. Torok-Storb. All cells were used within 8 passages after receipt or were authenticated by matching transcript profiles of the cells used in these experiments with transcript profiles generated from the cell stocks originally provided or from public gene expression datasets corresponding to the specific cell line in order to confirm the identity of the cell line. Epithelial CaP cells and Hs5 cells were maintained in RPMI 1640 with 10% FBS. Hs27a cells were maintained in DMEM with 10% FBS. PSC27 cells were grown and maintained as described earlier [14]. PSC27, Hs5 and Hs27a cells were transduced with viral particles when at 50% confluence in the presence of 6 μg/ml ‘Polybrene’. Transduced cells were selected for at least 14 days using 5 μg/ml of Blasticidin. Cells were analyzed for GDNF-V5 over-expression by both Western blot, using the V5-antibody, and by GDNF specific ELISA.

### Microarray Experiments and Data Analysis

To provide a reference standard RNA for use on two-color cDNA microarrays, we combined equal quantities of total RNA isolated from LNCaP, DU145, PC3, and CWR22 prostate epithelial cell lines grown at log phase, amplifyed through two rounds using the MessageAmp™ II aRNA Amplification Kit (Ambion®), and converting to first strand cDNA. RNA for the experimental conditions was purified using Trizol (Life Technologies, Rockville, MD) following the manufacturer's protocol followed by further purification by RNeasy kit (Qiagen Inc, Valencia, CA) including DNAse treatment using the RNase-Free DNase Set (Qiagen Inc, Valencia, CA). Total RNA from experimental samples were amplified two rounds using the Ambion MessageAmp aRNA Kit (Ambion Inc, Austin, TX), according to the manufacturer's specifications. cDNA probe pairs were prepared by aminoallyl reverse transcription using 2 μg of amplified RNA and labeling with Cy3-dCTP or Cy5-dCTP fluorescent dyes (Amersham Bioscience, Piscataway, NJ). Experimental and reference probes were combined and competitively hybridized to Agilent Human 4x44K microarrays under a coverslip for 16 h at 63°C. Fluorescent array images were collected for Cy3 and Cy5 emissions using a GenePix 4000B fluorescent scanner (Axon Instruments, Foster City, CA). Image intensity data were extracted and analyzed using GenePix Pro v4.1.1.44 software. Log2 ratios were normalized using the printtiploess function from the Limma package in R. The data-sets were uploaded to Ingenuity Pathway Analysis Software Platform for further analysis, including activation scores and pathway analysis.

### Patient Stromal Cell Isolation

Stromal cells were isolated from patient-matched tumor biopsies before and after chemotherapy treatment using an Arcturus (Veritas Microdissection) laser capture microscope. The criteria for selecting captured stromal cells were as follows: the stroma had to be adjacent to cancerous glands and epithelial cells to select for cancer associated stromal cells. Additionally, a safety margin of about 10 cells from the basal membrane was applied to prevent contamination with epithelial cells. The purity of stromal cells collected by LCM was later confirmed by qRT-PCR with stroma-specific markers and epithelium markers, respectively, with the former much higher than the latter (minimum of 15 cycles' difference). Approximately 3000 stromal cells were collected per sample. RNA isolation and array experiments were performed as described above.

## SUPPLEMENTARY MATERIAL, FIGURES


