# Supplementary material for: DNA damage induces GDNF secretion in the tumor microenvironment with paracrine effects promoting prostate cancer treatment resistance
Source: Oncotarget. 2014 Dec 10;6(4):2134–47. doi: 10.18632/oncotarget.3040 (PMC4385841; doi:10.18632/oncotarget.3040)
Supplement: Supplementary file 1 [file oncotarget-06-2134-s001.pdf]

DNA damage induces GDNF secretion in the tumor microenvironment with paracrine effects promoting prostate cancer treatment resistance

Supplementary Material

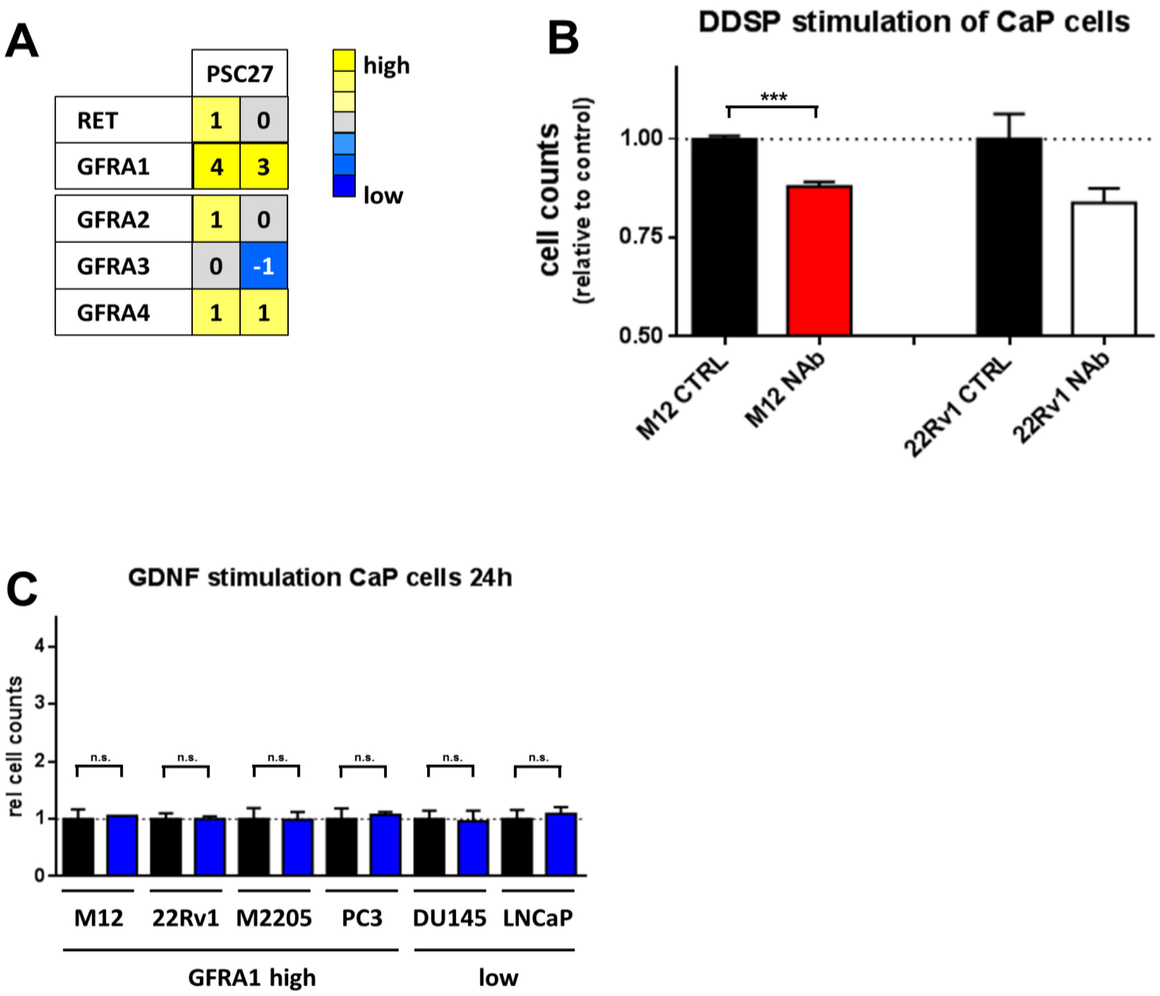

Figure S1:

(A) Microarray transcript level analysis for GDNF receptors in PSC27 cells. Transcript level analysis for RET receptor and GFRA-family members in PSC27 cells by microarray analysis. Each box represents one individual array probe averaged for 3 sample reads.

(B) GDNF neutralizing antibodies reduces the growth promoting effect of conditioned medium after PSC27 irradiation. Relative cell counts for M12 and 22Rv1 cells after 5 days stimulation with conditioned medium from irradiated PSC27 cells in the presence or absence of neutralizing anti-GDNF antibodies (Nab). M12 cells with GDNF Nab show reduced proliferation to DDSP CM by 12% ($p<0.001$) and 22Rv1 by 15% ($p=0.069$).

(C) GDNF stimulation of CaP epithelial cells for 24h as control for invasion assay. Cell proliferation assay counting viable cells after 24h of stimulation with 100 ng/ml hrGDNF in serum free conditions as control to invasion assay. Values are normalized to control conditions w/o GDNF per cell line.
